# Impacto Clínico da Avaliação da Reserva de Fluxo Miocárdico na Identificação da Causa do Desconforto Torácico

**DOI:** 10.36660/abc.20230700

**Published:** 2024-06-21

**Authors:** Ronaldo Lima, André Luiz Ferreira Bezerra, Marianna Daibes, Claudio Domenico, Andrea De Lorenzo

**Affiliations:** 1 Universidade Federal do Rio de Janeiro Rio de Janeiro RJ Brasil Universidade Federal do Rio de Janeiro, Rio de Janeiro, RJ – Brasil; 2 Instituto Nacional de Cardiologia Rio de Janeiro RJ Brasil Instituto Nacional de Cardiologia, Rio de Janeiro, RJ – Brasil

**Keywords:** Reserva de Fluxo Miocárdico, Isquemia Miocárdica, SPECT

## Abstract

**Fundamento:**

Gama-câmaras com detectores de telureto-cádmio-zinco (CZT) permitiram a quantificação da reserva de fluxo miocárdico (RFM), podendo aumentar a acurácia da cintilografia miocárdica de perfusão (CMP) para detectar a causa do desconforto torácico.

**Objetivo:**

Avaliar o impacto clínico da RFM para detectar a causa do desconforto torácico.

**Métodos:**

171 pacientes com desconforto torácico que foram submetidos a coronariografia ou angiotomografia de coronárias também realizaram CMP e RFM num intervalo de tempo <30 dias. As aquisições das imagens dinâmicas de repouso e estresse foram iniciadas simultaneamente à injeção de 99mTc sestamibi (10 e 30mCi, respectivamente), ambas com duração de onze minutos, seguidas imediatamente pela aquisição das imagens de perfusão durante 5 minutos. O estresse foi realizado com dipiridamol. Uma RFM global ou por território coronariano <2,0 foi classificada como anormal.

**Resultados:**

A idade média foi de 65,9±10 anos (60% do sexo feminino). A avaliação anatômica mostrou que 115 (67,3%) pacientes apresentavam obstrução coronariana significativa, sendo que, 69 apresentavam CMP anormal e 91 apresentavam RFM anormal (60,0% vs. 79,1%, p<0,01). Dentre os pacientes sem obstrução (56 – 32,7%), 7 tinham CMP anormais e 23 tinham RFM global reduzida. A realização da RFM identificou a etiologia do desconforto torácico em 114 pacientes enquanto a CMP identificou em 76 (66,7% vs. 44,4%, p<0,001).

**Conclusão:**

A RFM é uma medida fisiológica quantificável que aumenta o impacto clínico da CMP na detecção da causa do desconforto torácico através de uma maior acurácia para detecção de DAC obstrutiva e ainda possibilita identificar a presença de doença microvascular.

## Graphical abstract

**Figure:**
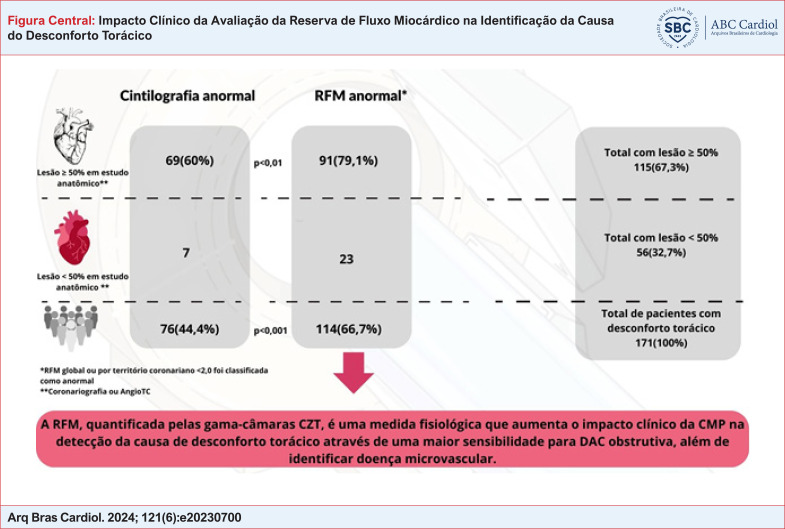

## Introdução

O desconforto torácico é uma queixa extremamente frequente na prática clínica cuja etiologia pode ser de difícil determinação, especialmente em grupos populacionais como mulheres, idosos e diabéticos, por exemplo. Ele pode ser agudo, quando de início novo ou com alteração abrupta de padrão, intensidade ou duração comparada com episódios prévios, ou estável, quando recorrente ou crônica e associada com fatores desencadeantes conhecidos e consistentes, como esforço físico ou estresse emocional. Apesar de o termo "dor" ser de uso frequente, a sensação pode ser diversa, como pressão, aperto, ardência, ou desconforto, e a localização pode ser cervical, epigástrica, nos ombros ou mandíbula.^1^

A cintilografia de perfusão miocárdica (CPM) com tomografia computadorizada por emissão de fóton único (SPECT) é importante para o diagnóstico e avaliação prognóstica de pacientes com doença arterial coronariana (DAC).^2^ Apesar de seus valores diagnóstico e prognóstico comprovados, a avaliação das imagens de perfusão é realizada comparando a captação relativa do radiofármaco nas diferentes paredes miocárdicas, o que pode limitar a capacidade da SPECT de identificar pacientes com DAC multiarterial de alto risco (obstruções ≥70% em duas ou mais coronárias epicárdicas, com lesões proximais, ou acometimento do tronco da coronária esquerda, artéria descendente anterior proximal, grande área de miocárdio sob risco).^3^

Essa limitação pode ser superada pela quantificação do fluxo sanguíneo miocárdico (FSM) ou da reserva de fluxo miocárdico (RFM), usando a cinética dos traçadores na tomografia por emissão de pósitrons (PET).^4,5^ A PET é um método não invasivo bem validado para quantificação da perfusão miocárdica, demonstrando um poder incremental de diagnóstico e prognóstico quando comparada à CPM em pacientes com suspeita ou DAC conhecida.^6–10^ Além disso, a RFM permite identificar a presença de doença microvascular como causa da angina em pacientes com artérias coronárias "normais" avaliadas pela coronariografia ou angiotomografia.^11^

Câmaras de cádmio-zinco-telureto (CZT) de alta sensibilidade, dedicadas a exames cardiológicos, permitem a aquisição dinâmica de imagens tomográficas adequadas para avaliação da cinética do radiotraçador e abrem uma nova era para a quantificação do FSM e da RFM.^12^

Todavia, como se trata de uma técnica nova e em crescimento, o impacto clínico do emprego da avaliação da RFM medida através de gama-câmara CZT na investigação de etiologia isquêmica do desconforto torácico ainda é desconhecido. Desta forma, este estudo procurou avaliar os resultados do uso da quantificação da RFM em pacientes em investigação de desconforto torácico, comparando-os com o uso da CPM convencional na definição da presença de alterações de fluxo sanguíneo coronariano.

## Métodos

### População

Foram estudados 171 pacientes adultos, encaminhados para CPM pelos seus médicos assistentes para avaliação diagnóstica de desconforto torácico. Todos os pacientes estavam clinicamente estáveis e foram submetidos a angiografia coronária invasiva ou angiotomografia de coronárias (CTCA) nos 30 dias anteriores à CPM.

Os critérios de exclusão incluíram contraindicações para estresse farmacológico com dipiridamol, índice de massa corporal ≥ 40 kg/m^2^, insuficiência cardíaca (classes III/IV da New York Heart Association), síndrome coronariana aguda até 30 dias antes da inclusão no estudo, intervenções coronárias entre os exames para determinação da anatomia coronária e a CPM, e gravidez.

O estudo envolvendo participantes humanos foi aprovado pela Comitê de Ética do Hospital Clementino Fraga Filho da UFRJ. Os pacientes forneceram seu consentimento informado por escrito para participar deste estudo. O consentimento informado por escrito foi obtido do(s) indivíduo(s) para a publicação de quaisquer imagens ou imagens potencialmente identificáveis ou dados incluídos neste artigo.

### Avaliação da anatomia coronária

Os pacientes foram submetidos a angiografia coronária invasiva (69- 40,35%) ou CTCA (102 - 59,65%) usando técnicas padrão e no máximo 30 dias antes de realizarem a CPM/RFM. Para angiografia coronária, dois cardiologistas intervencionistas experientes classificaram as lesões estenóticas visualmente como porcentagem da estenose do diâmetro luminal. Uma lesão obstrutiva significativa foi classificada como >50% em uma artéria epicárdica importante. Os vasos que apresentavam lesões múltiplas foram classificados com base no maior grau de estenose. Os estudos CTCA foram realizados em um scanner de 128 cortes (Revolution HD, GE Healthcare, EUA) com disparo prospectivo de eletrocardiograma. Dois observadores experientes classificaram as lesões estenóticas visualmente como porcentagem da estenose do diâmetro luminal. Uma lesão obstrutiva significativa foi classificada como >50% em uma artéria epicárdica importante. Os vasos que apresentavam lesões múltiplas foram classificados com base no maior grau de estenose.

### Protocolo de estudo

Os pacientes foram submetidos a protocolo de um dia, com fase de repouso seguida de estresse farmacológico com dipiridamol. Eles foram instruídos a se abster de cafeína, substâncias contendo metilxantinas e fumar por 24 horas antes do exame. Medicamentos adicionais foram mantidos a critério dos médicos solicitantes. As varreduras foram realizadas em uma gama-câmara com colimador multi-pinhole e detectores estacionários pixelados de estado sólido feitos de telureto de cádmio-zinco (Discovery 530, GE Healthcare, Milwaukee, EUA) com 99mTc-sestamibi como radiotraçador conforme protocolo descrito previamente.^13^ Para permitir o posicionamento do coração no campo de visão da câmera, uma dose teste (18,5 MBq) foi administrada para uma pré-varredura de 60 segundos. A aquisição dinâmica de repouso no modo lista foi iniciada simultaneamente à injeção intravenosa manual de 30 segundos de 99mTc-sestamibi, na dose de 370 MBq, seguida de injeção de solução salina por 30 segundos, e durou 11 minutos com o paciente posicionado em decúbito dorsal. Imagens de perfusão em repouso foram obtidas imediatamente após a aquisição dinâmica por 5 minutos. Com o paciente ainda posicionado dentro da câmara, foi realizada injeção intravenosa de dipiridamol na dose de 0,14 mg/kg/min por 4 minutos, sob monitorização eletrocardiográfica. No pico do estresse, foi administrada uma segunda dose do radiotraçador (1.110 MBq) em 30 segundos, simultaneamente ao início da aquisição do estresse dinâmico, também com duração de 11 minutos. Da mesma forma, imagens de perfusão na posição supina foram obtidas imediatamente após a fase de estresse dinâmico, por 3 minutos. A aminofilina foi injetada 11 minutos após o início do estresse farmacológico em todos os pacientes. Imagens de estresse em posição prona foram obtidas em todos os pacientes com duração de 2 minutos.

Os dados estáticos e dinâmicos foram processados utilizando uma estação de trabalho dedicada (Xeleris 4.0, GE Healthcare, Haifa, Israel) e software disponível comercialmente (Corridor4DM, INVIA Medical Imaging Solutions, Ann Arbor, Michigan, EUA). As imagens dinâmicas do modo de lista foram reorganizadas em 22 quadros, consistindo nos primeiros 18 quadros de 10 segundos (180 segundos) e quatro quadros de 120 segundos (480 segundos). As imagens foram reconstruídas utilizando um algoritmo iterativo de maximização de expectativa de máxima verossimilhança (MLEM), com pós-filtro 3D do tipo Butterworth, sem atenuação ou correção de dispersão. Os contornos do ventrículo esquerdo (VE) foram gerados automaticamente a partir de imagens miocárdicas somadas de 2 minutos até o final da aquisição e uma região de interesse 3D (ROI) no meio do VE foi usada para amostrar a atividade do "pool" sanguíneo. A captação miocárdica foi estimada utilizando um modelo de retenção líquida generalizada.^14,15^ O transbordamento do miocárdio para o reservatório de sangue foi definido como zero, pois já foi descrito como insignificante.^16^ O FSM foi calculado usando um modelo de fluxo para Tc-99m^14^ e a RFM foi calculada como a razão entre o FSM de estresse e o em repouso. A subtração da atividade residual de repouso da série dinâmica de estresse foi realizada conforme descrito anteriormente.^13^ Os resultados foram relatados global e regionalmente, como três regiões vasculares ou regiões de mapas polares de 17 segmentos. A correção de movimento foi realizada para cada quadro quando apropriado. No presente estudo, o ponto de corte escolhido para RFM foi 2,0 conforme validado anteriormente.^17^

Foi realizada interpretação visual semiquantitativa utilizando um modelo de 17 segmentos. Os segmentos foram pontuados usando um sistema padrão de cinco pontos e foram obtidos o escore de estresse somado (SSS), o escore somado de repouso (SRS) e o escore de diferenças somadas (SDS). Um estudo anormal foi considerado quando o SSS foi >3.^13^ Foi avaliada a presença de isquemia miocárdica (SDS>1)^12^ em cada território vascular. Para efeitos deste estudo, dois especialistas determinaram cegamente o envolvimento de diferentes territórios coronários. A fração de ejeção do ventrículo esquerdo (FEVE) foi calculada automaticamente utilizando software disponível comercialmente (QGS, Cedars-Sinai Medical Center, Los Angeles, EUA).

### Análise estatística

Devido às características do estudo (estudo exploratório), não foi realizado cálculo de tamanho amostral, sendo o presente grupo de pacientes uma amostra de conveniência.

A normalidade das variáveis foi avaliada através do teste de Kolmogorov-Smirnov. Variáveis contínuas com distribuição normal foram expressas como média ± desvio padrão (DP) e variáveis categóricas como número e percentual (%). A presença de uma CPM com isquemia, RFM anormal (<2,0) ou CAT ou CTCA com obstrução coronária >50% foram indicadas como causa de dor precordial.

Ao analisar diferenças entre dois grupos, aplicamos o teste t independente ao comparar variáveis contínuas (RFM) e o teste η^2^ ou o teste exato de Fisher, conforme apropriado quando comparação de variáveis categóricas (presença de CPM anormal).

Um nível de significância de 5% foi adotado em todas as análises.

As análises foram realizadas no programa SPSS versão 20.0 (IBM Estatísticas, Armonk, NY, Estados Unidos).

## Resultados

A idade média da população foi de 65,9±10 anos e 60% dos pacientes eram do sexo feminino. Hipertensão, dislipidemia e diabetes foram os fatores de risco mais frequentes. As características basais da população estudada são mostradas na Tabela 1

**Tabela 1:** Características Demográficas (171 pacientes)

Idade	65,9±10 anos
Sexo Feminino	103 (60,2%)
IMC	29,5±5,7
Hipertensão	139 (81,3%)
Diabetes	69 (40,4%)
Dislipidemia	69 (40,4%)
Tabagismo	22 (12,9%)
História Familiar	58 (33,9%)

IMC: índice de massa corporal.

Em relação aos dados da CPM, o SSS e SDS foram menores entre os pacientes com lesão obstrutiva significativa assim a RFM. Os resultados da cintilografia e da avaliação do fluxo podem ser observados na Tabela 2.

**Tabela 2:** Parâmetros cintilográficos e de avaliação de fluxo

	Total	Com lesão>50%	Sem lesão >50%	Valor de p
SSS	5,14 ± 5,95	6,27 ± 7,24	3,64 ± 4,46*	0,028
SRS	2,68 ± 4,76	3,11 ± 5,73	2,06 ± 3,76	0,271
SDS	2,45 ± 2,95	2,97 ± 3,22	1,83 ± 2,55*	0,044
FEVE	60,6 ± 12,0	59,5 ± 12	62,0 ± 11,5*	0,044
FME ml/g/min	1,56 ± 0,68	1,50 ± 0,69	1,62 ± 0,74	0,366
FMR ml/g/min	0,65 ± 0,28	0,69 ± 0,32	0,61 ± 0,24	0,199
RFM	2,49 ± 0,93	2,27 ± 0,85	2,68 ± 0,94*	0,019

FEVE: fração de ejeção do ventrículo esquerdo; FME: fluxo miocárdico de estresse; FMR: fluxo miocárdico de repouso; RFM: reserva de fluxo miocárdico; SDS: escore somado das diferenças; SRS: escore somados de repouso; SSS: escore somados de estresse;

* =p<0,05.

**Figura 1:**
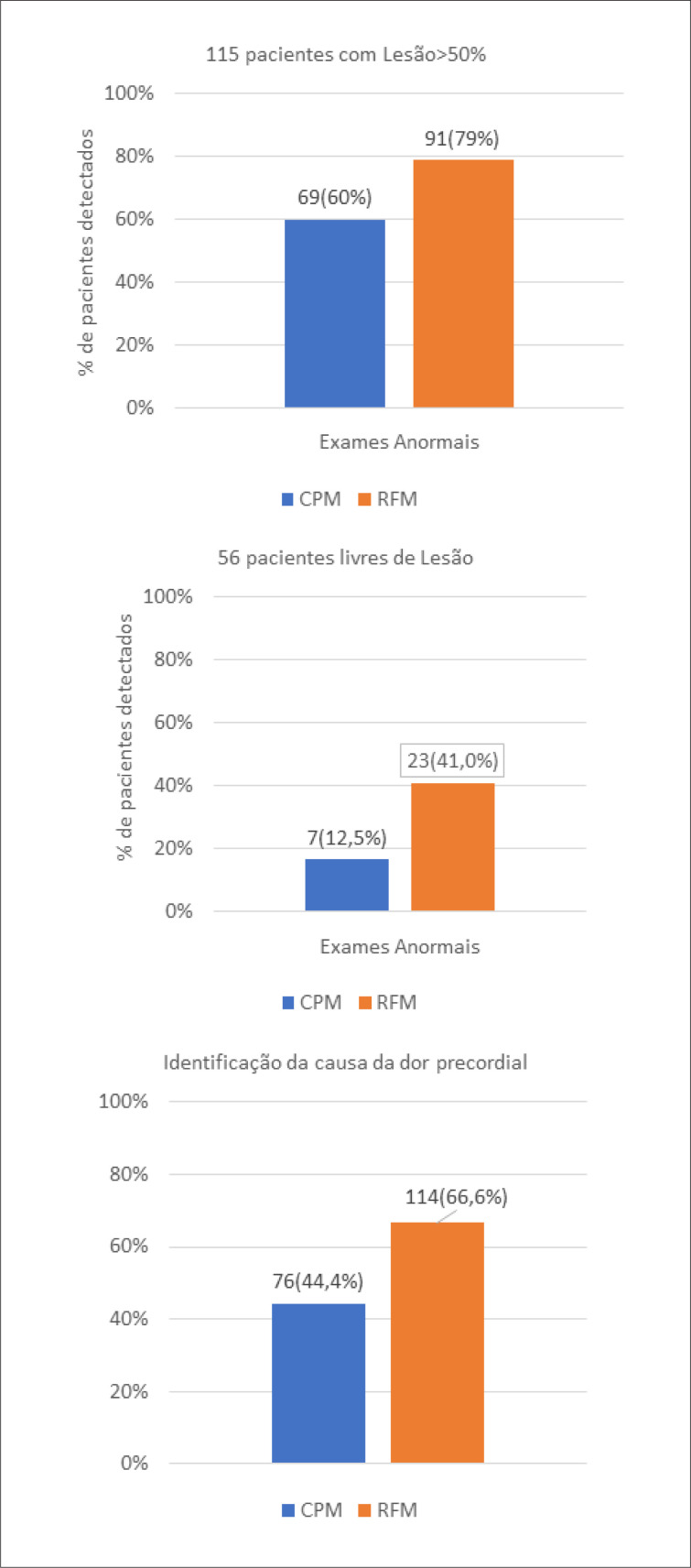
Identificação da causa de desconforto torácico pela cintilografia miocárdica e pela reserva de fluxo na população estudada, naqueles com ou sem lesão coronariana obstrutiva. CPM: cintilografia de perfusão miocárdica; RFM: reserva de fluxo miocárdico.

**Figura 2:**
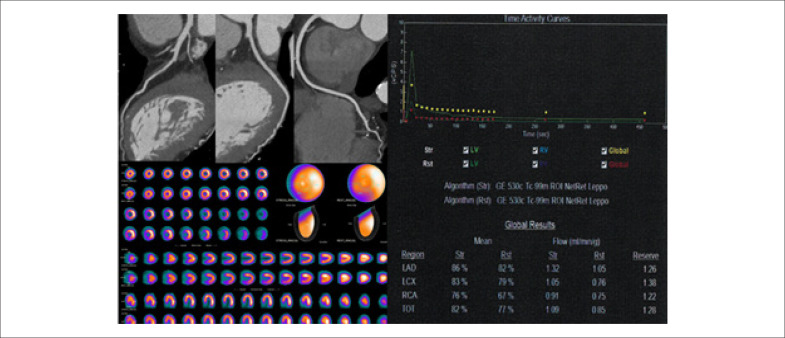
Mulher, 78 anos, hipertensa e dislipidêmica com dor precordial típica há 2 anos. Cintilografia com dipiridamol foi negativa para isquemia há 1 ano. Angio TC recente normal. Avaliação da reserva de fluxo miocárdico global e territoriais diminuídas.

Dos 115 pacientes que apresentavam obstrução significativa, 69 tinham CPM com defeito reversível e 91 apresentavam RFM reduzida (60,0% vs 79,1%, p<0,01). Os achados dos estudos anatômicos estão demonstrados na Tabela 3. Dentre aqueles em que não se observou alterações coronarianas significativas no exame anatômico (56 – 32,7%),^7^ (12,5%) tinham CMP anormais e 23 (41%) tinham RFM global reduzida (Figura 1). Dessa forma, a RFM pôde ser associada com a dor cardíaca de etiologia isquêmica em 114 pacientes (66,6%) e a CPM em 76 (44,4%) (Figura 2).

**Tabela 3:** Resultado dos exames de anatomia coronariana

	Sem lesão coronariana	1 vaso com obstrução	2 vasos com obstrução	3 vasos com obstrução
CTCA - 102	35 (34,3%)	30 (28,9%)	29 (31,6%)	8 (7,8%)
CAT - 69	21 (30,4%)	21 (30,4%)	21 (30,4%)	6 (8,8%)

Angio TC: angiotomografia coronariana; CAT: cateterismo cardíaco.

## Discussão

Em pacientes com desconforto torácico, o percentual de doença coronariana obstrutiva como causa vem diminuindo. No estudo de Patel, menos de 50% com angina tinham lesão coronariana significativa.^17^ Estudos com tomografia de emissão de pósitrons mostram que a alteração de RFM pode, além de aumentar a sensibilidade para detectar DAC, identificar a doença microvascular como causa de desconforto torácico.^11^

As gama-câmaras com detectores de CZT vêm se revelando uma alternativa para avaliação de RFM. O estudo de Souza et al.^13^ revelou que essa técnica é factível e o estudo de Lima et al.^17^ demonstrou uma acurácia maior na detecção de doença coronariana obstrutiva.

No presente estudo, observamos que o desconforto torácico foi associado a doença coronariana obstrutiva significativa em 67,3%, percentual maior do que o estudo de Patel et al.,^18^ mas semelhante à observada no estudo de Gerber et al.^19^

Conforme demonstrado anteriormente, o uso da RFM aumenta a acurácia da CPM para detecção de DAC obstrutiva. No estudo de Lima et al.,^17^ a sensibilidade foi de 55,2% e 69% da CPM e da RFM respectivamente. No presente estudo foi de 60% e 79,1%, mas por paciente e não por vaso como analisado no estudo anterior.

Neste estudo, 41% dos pacientes sem DAC obstrutiva revelaram alteração de RFM. Esses valores são semelhantes aos do estudo CORMICA no qual metade dos pacientes com angina e coronárias normais apresentavam doença da microcirculação através da avaliação invasiva com teste de adenosina.^20^ Já numa revisão sistemática sobre pacientes com angina e coronária normais, 30% dos pacientes apresentaram RFM anormal.^11^

Como pudemos observar neste estudo e em outros, a avaliação anatômica é cada vez menos eficiente para determinar a causa do desconforto precordial sendo cada vez mais necessária a avaliação da RFM ou a identificação de vasoespasmo coronariano. Embora não fosse o objetivo deste estudo comparar testes anatômicos e funcionais, o percentual de pacientes que tiveram a etiologia isquêmica como causa do sintoma foi semelhante quando a análise da RFM foi acrescentada (Figura Central).

Por fim, este estudo sugere que na impossibilidade de realizar a avaliação da RFM com PET, por falta do equipamento ou dos traçadores necessários, as gama-câmaras com detectores de CZT mostram-se uma excelente alternativa.

### Limitações do estudo

A principal limitação deste estudo é que não podemos afirmar que a presença de uma RFM anormal seja a causa do desconforto precordial, da mesma forma que a presença de uma CPM anormal ou uma lesão obstrutiva coronariana. De qualquer forma, a presença de uma RFM reduzida corresponde a um dos critérios determinados pelo grupo COVADIS para angina microvascular.^21^ Outra limitação é que todos os participantes foram encaminhados para exames anatômicos para diagnóstico de DAC obstrutiva o que seria um viés importante de seleção. Entretanto como o uso da CTCA como exame diagnóstico tem se tornado cada vez mais frequente pode ter minimizado esse fato. Finalmente, este é um estudo unicêntrico e outras investigações, especialmente multicêntricas, podem ajudar a confirmar os resultados encontrados.

## Conclusões

A avaliação da RFM é útil para auxiliar na identificação da etiologia do desconforto precordial, mostrando-se superior à CPM com avaliação convencional da perfusão miocárdica. A possibilidade de uso da gama-câmara CZT para medida da RFM permite facilitar o emprego da técnica, para além do PET, ampliando seu uso, com potenciais benefícios diagnósticos para pacientes com dor precordial a esclarecer.
