# Supplementary material for: Impacto Clínico da Avaliação da Reserva de Fluxo Miocárdico na Identificação da Causa do Desconforto Torácico
Source: Arq Bras Cardiol. 2024 Jun 21;121(6):e20230700. [Article in Portuguese] doi: 10.36660/abc.20230700 (PMC11216331; doi:10.36660/abc.20230700)
Supplement: Translation

# Translation: Clinical Impact of Assessment of Myocardial Flow Reserve in Identifying the Cause of Chest Discomfort

**DOI:** 10.36660/abc.20230700i

**Authors:** Ronaldo Lima, André Luiz Ferreira Bezerra, Marianna Daibes, Claudio Domenico, Andrea De Lorenzo

**Affiliations:** 1 Rio de Janeiro RJ Brazil Universidade Federal do Rio de Janeiro, Rio de Janeiro, RJ – Brazil; 2 Rio de Janeiro RJ Brazil Instituto Nacional de Cardiologia, Rio de Janeiro, RJ – Brazil

**Keywords:** Myocardium Flow Reserve, Myocardium Ischemia, SPECT

## Abstract

**Background:**

Gamma cameras with cadmium-zinc telluride (CZT) detectors allowed the quantification of myocardial flow reserve (MBF), which can increase the accuracy of myocardial perfusion scintigraphy (MPS) to detect the cause of chest discomfort.

**Objective:**

To assess the clinical impact of MBF to detect the cause of chest discomfort.

**Methods:**

171 patients with chest discomfort who underwent coronary angiography or coronary CT angiography also underwent MPS and MBF in a time interval of <30 days. The acquisitions of dynamic imaging of rest and stress were initiated simultaneously with the 99mTc injection sestamibi (10 and 30mCi, respectively), both lasting eleven minutes, followed by immediately acquiring perfusion images for 5 minutes. The stress was performed with dipyridamole. A global or per coronary territory MBF <2.0 was classified as abnormal.

**Results:**

The average age was 65.9±10 years (60% female). The anatomical evaluation showed that 115 (67.3%) patients had coronary obstruction significant, with 69 having abnormal MPs and 91 having abnormal MBF (60.0% vs 79.1%, p<0.01). Among patients without obstruction (56 – 32.7%), 7 had abnormal MPS, and 23 had reduced global MBF. Performing MBF identified the etiology of the chest discomfort in 114 patients while MPS identified it in 76 (66.7% vs 44.4%, p<0.001).

**Conclusion:**

MBF is a quantifiable physiological measure that increases the clinical impact of MPS in detecting the cause of chest discomfort through greater accuracy for detecting obstructive CAD, and it also makes it possible to identify the presence of the microvascular disease.

## Introduction

Chest discomfort is an extremely common complaint in clinical practice, and its etiology can be difficult to determine, especially in population groups such as women, the elderly, and diabetics, for example. It can be acute, when new in onset or with an abrupt change in pattern, intensity, or duration compared to previous episodes, or stable, when recurrent or chronic, and associated with known and consistent triggering factors, such as physical effort or emotional stress. Although the term "pain" is frequently used, the sensation can be diverse, such as pressure, tightness, burning, or discomfort, and the location can be cervical, epigastric, in the shoulders, or jaw.^1^

Myocardial perfusion scintigraphy (MPS) with single photon emission computed tomography (SPECT) is important for the diagnosis and prognostic evaluation of patients with coronary artery disease (CAD).^2^ Despite its proven diagnostic and prognostic values, the evaluation of perfusion imaging is performed by comparing the relative uptake of the radiopharmaceutical in the different myocardial walls, which may limit the ability of SPECT to identify patients with high-risk multivessel CAD (obstructions ≥70% in two or more epicardial coronary arteries, with proximal lesions, or involvement of the left coronary trunk, proximal anterior descending artery, large area of myocardium at risk).^3^

This limitation can be overcome by quantifying myocardial blood flow (MBF) or myocardial flow reserve (MFR), using tracer kinetics in positron emission tomography (PET).^4,5^ PET is a well-established non-invasive method, validated for quantifying myocardial perfusion, demonstrating an incremental diagnostic and prognostic power when compared to MPS in patients with suspected or known CAD.^6–10^ Furthermore, MBF allows identifying the presence of microvascular disease as the cause of angina in patients with "normal" coronary arteries assessed by coronary angiography or computed tomography angiography.^11^

High-sensitivity cadmium-zinc-telluride (CZT) chambers, dedicated to cardiological examinations, allow the dynamic acquisition of tomographic images suitable for evaluating radiotracer kinetics and open a new era for the quantification of MBF and MFR.^12^

However, as this is a new and growing technique, the clinical impact of using the assessment of MFR, measured using a CZT gamma camera, in investigating the ischemic etiology of chest discomfort is still unknown. Therefore, this study sought to evaluate the results of using MBF quantification in patients undergoing investigation of chest discomfort, comparing them with the use of conventional MPS in defining the presence of changes in coronary blood flow.

## Methods

### Population

One hundred and seventy-one adult patients were studied, referred to MPS by their attending physicians for diagnostic evaluation of chest discomfort. All patients were clinically stable and underwent invasive coronary angiography or coronary CT angiography (CTCA) within 30 days before MPS.

Exclusion criteria included contraindications to pharmacological stress with dipyridamole, body mass index ≥ 40 kg/m^2^, heart failure (New York Heart Association classes III/IV), acute coronary syndrome within 30 days before study inclusion, coronary interventions between exams to determine coronary anatomy and MPS, and pregnancy.

The study involving human participants was approved by the Ethics Committee of the Clementino Fraga Filho Hospital at UFRJ. Patients provided their written informed consent to participate in this study. Written informed consent was obtained from the individual(s) for the publication of any potentially identifiable images or images or data included in this article.

### Assessment of coronary anatomy

Patients underwent invasive coronary angiography (69 - 40.35%) or CCTA (102 - 59.65%) using standard techniques and a maximum of 30 days before performing MPS/MBF. For coronary angiography, two experienced interventional cardiologists classified stenotic lesions visually as a percentage of luminal diameter stenosis. A significant obstructive lesion was classified as >50% in a major epicardial artery. Vessels that had multiple lesions were classified based on the highest degree of stenosis. CCTA studies were performed on a 128-slice scanner (Revolution HD, GE Healthcare, USA) with prospective electrocardiogram (ECG) triggering. Two experienced observers classified stenotic lesions visually as a percentage of luminal diameter stenosis. A significant obstructive lesion was classified as >50% in a major epicardial artery. Vessels that had multiple lesions were classified based on the highest degree of stenosis.

### Study protocol

Patients underwent a 1-day protocol, with a rest phase followed by pharmacological stress with dipyridamole. They were instructed to abstain from caffeine, substances containing methylxanthines, and smoking for 24 hours before the examination. Additional medications were maintained at the discretion of the requesting physicians. The scans were performed on a gamma camera with a multi-pinhole collimator and stationary solid-state pixelated detectors made of cadmium-zinc telluride (Discovery 530, GE Healthcare, Milwaukee, USA) with 99mTc-sestamibi as radiotracer according to the previously described protocol.^13^ To allow positioning of the heart in the camera's field of view, a test dose (18.5 MBq) was administered for a 60-second prescan. The resting dynamic acquisition in list mode was initiated simultaneously with a manual intravenous injection of 30 seconds of 99mTc-sestamibi, at a dose of 370 MBq, followed by an injection of saline solution for 30 seconds, and lasted 11 minutes with the patient positioned in the supine position. Resting perfusion images were obtained immediately after dynamic acquisition for 5 minutes. With the patient still positioned inside the camera, an intravenous injection of dipyridamole was performed at a dose of 0.14 mg/kg/min for 4 minutes, under electrocardiographic monitoring. At the peak of stress, a second dose of radiotracer (1,110 MBq) was administered within 30 seconds, simultaneously with the beginning of dynamic stress acquisition, also lasting 11 minutes. Likewise, perfusion images in the supine position were obtained immediately after the dynamic stress phase, for 3 minutes. Aminophylline was injected 11 minutes after the onset of pharmacological stress in all patients. Prone stress images were obtained in all patients lasting 2 minutes.

Static and dynamic data were processed using a dedicated workstation (Xeleris 4.0, GE Healthcare, Haifa, Israel) and commercially available software (Corridor4DM, INVIA Medical Imaging Solutions, Ann Arbor, Michigan, USA). The list mode dynamic images were rearranged into 22 frames, consisting of the first 18 frames of 10 seconds (180 seconds) and four frames of 120 seconds (480 seconds). Images were reconstructed using an iterative maximum likelihood expectation maximization (MLEM) algorithm, with a Butterworth-type 3D post-filter, without attenuation or scatter correction. Left ventricular (LV) contours were automatically generated from summed myocardial images from 2 minutes until the end of acquisition and a 3D region of interest (ROI) in the middle of the LV was used to sample blood pool activity. Myocardial uptake was estimated using a generalized fluid retention model.^14,15^ Myocardial overflow into the blood reservoir was set to zero as it has previously been described as negligible.^16^ The MBF was calculated using a flow model for Tc-99m^14^ and the MFR was calculated as the ratio between the stress and rest MBF. Subtraction of resting residual activity from the dynamic stress series was performed as previously described.^13^ Results were reported globally and regionally, as three vascular regions or 17-segment polar map regions. Motion correction was performed for each frame when appropriate. In the present study, the cutoff point chosen for MBF was 2.0 as previously validated.^17^

A semiquantitative visual interpretation was performed using a 17-segment model. Segments were scored using a standard five-point system and summed stress score (SSS), summed rest score (SRS), and summed difference score (SDS) were obtained. An abnormal study was considered when the SSS was >3.^13^ The presence of myocardial ischemia (SDS>1)^12^ was assessed in each vascular territory. For this study, two experts blindly determined the involvement of different coronary territories. Left ventricular ejection fraction (LVEF) was calculated automatically using commercially available software (QGS, Cedars-Sinai Medical Center, Los Angeles, USA).

### Statistical analysis

Due to the characteristics of the study (exploratory study), no sample size calculation was performed, with the present group of patients being a convenience sample.

The normality of the variables was assessed using the Kolmogorov-Smirnov test. Continuous variables with normal distribution were expressed as mean ± standard deviation (SD) and categorical variables as number and percentage (%). The presence of a CPM with ischemia, abnormal RFM (<2.0), or CAT or CTCA with coronary obstruction >50% were indicated as the cause of precordial pain.

When analyzing differences between the two groups, we applied an independent t-test when comparing continuous variables (RFM) and η^2^ test or a Fisher's exact test, as appropriate when comparing categorical variables (presence of abnormal CPM).

A significance level of 5% was adopted in all analyses.

Analyses were performed using SPSS version 20.0 (IBM Statistics, Armonk, NY, United States).

## Results

The average age of the population was 65.9±10 years and 60% of patients were female. Hypertension, dyslipidemia, and diabetes were the most frequent risk factors. The baseline characteristics of the studied population are shown in Table 1.

**Table 1:** Demographic Characteristics (171 patients)

Age	65.9±10 years
Female	103 (60.2%)
BMI	29.5±5.7
Hypertension	139 (81.3%)
Diabetes	69 (40.4%)
Dyslipidemia	69 (40.4%)
Smoking	22 (12.9%)
Family History	58 (33.9%)

BMI: body mass index.

Concerning MPS data, the SSS and the SDS were lower among patients with significant obstructive lesions. The results of the MPS and flow assessment can be seen in Table 2.

**Table 2:** Scintigraphic and flow assessment parameters

	Total	Lesion>50%	Lesion<50%	p Value
SSS	5.14 ± 5.95	6.27 ± 7.24	3.64 ± 4.46	0.028
SRS	2.68 ± 4.76	3.11 ± 5.73	2.06 ± 3.76	0.271
SDS	2.45 ± 2.95	2.97 ± 3.22	1.83 ± 2.55	0.044
LVEF	60.6 ± 12.0	59.5 ± 12	62.0 ± 11.5	0.044
SMF ml/g/min	1.56 ± 0.68	1.50 ± 0.69	1.62 ± 0.74	0.366
RMF ml/g/min	0.65 ± 0.28	0.69 ± 0.32	0.61 ± 0.24	0.199
MFR	2.49 ± 0.93	2.27 ± 0.85	2.68 ± 0.94	0.019

LVEF: left ventricle ejection fraction; MFR: myocardial flow reserve; RMF: rest myocardial flow; SMF: stress myocardial flow; SDS: summed difference score; SRS: summed resting score; SSS: summed stress score.

Of the 115 patients who had significant obstruction, 69 had MPS with a reversible defect and 91 had reduced MFR (60.0% vs 79.1%, p<0.01). The findings of the anatomical studies are shown in Table 3. Among those in which no significant coronary changes were observed in the anatomical examination (56 – 32.7%),^7^ (12.5%) had abnormal MPS and 23 (41%) had reduced global MFR (Figure 1). Thus, MFR could be associated with cardiac pain of ischemic etiology in 114 patients (66.6%) and MPS in 76 (44.4%). (Figure 2)

**Table 3:** Result of coronary anatomy exams

	No coronary lesion	One vessel disease	Two vessel disease	Three vessel disease
CCTA - 102	35 (34.3%)	30 (28.9%)	29 (31.6%)	8 (7.8%)
CATH- 69	21 (30.4%)	21 (30.4%)	21 (30.4%)	6 (8.8%)

CCTA: coronary computed tomography angiography; CATH: cardiac catheterization.

**Figure 1:** Identification of the cause of chest discomfort by myocardial scintigraphy and flow reserve in the studied population, in those with or without obstructive coronary lesion. MFR: myocardial flow reserve; MPS: Myocardial perfusion scintigraphy.

**Figure 2:** Woman, 78 years old, hypertensive and dyslipidemic with typical chest pain for 2 years. Dipyridamole scintigraphy was negative for ischemia 1 year ago. Recent normal CT angiography. Presence of decreased global and territorial myocardial flow reserve.

## Discussion

In patients with chest discomfort, the percentage of obstructive coronary disease as its cause has been decreasing. In the study by Patel et al, less than 50% of patients with angina had significant coronary lesions.^17^ Studies with positron emission tomography show that changes in MFR can, in addition to increasing sensitivity to detect CAD, identify microvascular disease as the cause of chest discomfort.^11^

Gamma cameras with CZT detectors have proven to be an alternative for assessing MFR The study by Souza et al.^13^ revealed that this technique is feasible and the study by Lima et al.^17^ demonstrated greater accuracy in detecting obstructive coronary disease.

In the present study, we observed that chest discomfort was associated with significant obstructive coronary disease in 67.3%, a higher percentage than the study by Patel et al.^18^ but similar to that observed in the study by Gerber et al.^19^

As previously demonstrated, the use of MFR increases the accuracy of MPS for detecting obstructive CAD. In the study by Lima et al.,^17^ the sensitivity was 55.2% and 69% of the MPS and MFR respectively. In the present study it was 60% and 79.1%, but per patient and not per vessel as analyzed in the previous study.

In this study, 41% of patients without obstructive CAD revealed changes in MFR. These values are similar to those of the CORMICA study in which half of the patients with angina and normal coronary arteries had microcirculation disease detected by invasive evaluation with an adenosine test.^20^ In a systematic review of patients with angina and normal coronary arteries, 30% of the patients presented with abnormal MFR.^11^

As we have seen in this study and others, anatomical assessment is less efficient in determining the cause of chest discomfort, making it increasingly necessary to assess MFR or identify coronary vasospasm. Although the comparison of anatomic and functional tests was not the objective of this study, the percentage of patients who had ischemic etiology as the cause of the symptom was similar when the MFR analysis was added (Central Illustration).

Finally, this study suggests that if it is impossible to assess MFR with PET, due to lack of the necessary equipment or tracers, gamma cameras with CZT detectors are an excellent alternative.

### Study limitations

The main limitation of this study is that we cannot state that the presence of an abnormal MFR is the cause of chest discomfort, in the same way as the presence of an abnormal MPS or a coronary obstructive lesion. In any case, the presence of a reduced MFR corresponds to one of the criteria determined by the COVADIS group for microvascular angina.^21^ Another limitation is that all participants were referred for anatomical examinations to diagnose obstructive CAD, which would be an important selection bias. However, as the use of CCTA as a diagnostic test has become increasingly frequent, this fact may have been minimized. Finally, this is a single-center study, and other investigations, especially multicenter ones, can help confirm the results found.

## Conclusions

The assessment of MFR is useful to help identify the etiology of chest discomfort, proving to be superior to MPS with conventional assessment of myocardial perfusion. The possibility of using the CZT gamma camera to measure MFR makes it easier to use the technique, in addition to PET, expanding its use, with potential diagnostic benefits for patients with chest pain to be clarified.
